# Combined genotype and haplotype distributions of *MTHFR* C677T and A1298C polymorphisms: A cross-sectional descriptive study of 13,473 Chinese adult women

**DOI:** 10.1097/MD.0000000000005355

**Published:** 2016-12-02

**Authors:** Shujun Fan, Boyi Yang, Xueyuan Zhi, Yanxun Wang, Quanmei Zheng, Guifan Sun

**Affiliations:** a Research Center of Environment and Non-Communicable Disease, School of Public Health, China Medical University, Shenyang; b Guangzhou Key Laboratory of Environmental Pollution and Health Risk Assessment, Department of Preventive Medicine, School of Public Health, Sun Yat-sen University, Guangzhou; c Division of Molecular Preventive Medicine, Shanghai Institute of Targeted Therapy and Molecular Medicine, Shanghai, China.

**Keywords:** A1298C, C677T, China, haplotype, methylenetetrahydrofolate reductase

## Abstract

Methylenetetrahydrofolate reductase (*MTHFR*) C677T and A1298C polymorphisms are, independently and/or in combination, associated with many disorders. However, data on the combined genotype and haplotype distributions of the 2 polymorphisms in Chinese population were limited.

We recruited 13,473 adult women from 9 Chinese provinces, collected buccal cell samples, and determined genotypes, to estimate the combined genotype and haplotype distributions of the *MTHFR* C677T and A1298C polymorphisms.

In the total sample, the 6 common combined genotypes were CT/AA (29.5%), TT/AA (21.9%), CC/AA (15.4%), CC/AC (14.9%), CT/AC (13.7%), and CC/CC (3.4%); the 3 frequent haplotypes were 677T-1298A (43.6%), 677C-1298A (37.9%), and 677C-1298C (17.6%). Importantly, we observed that there were 51 (0.4%) individuals with the CT/CC genotype, 92 (0.7%) with the TT/AC genotype, 17 (0.1%) with the TT/CC genotype, and that the frequency of the 677T-1298C haplotype was 0.9%. In addition, the prevalence of some combined genotypes and haplotypes varied among populations residing in different areas and even showed apparent geographical gradients. Further linkage disequilibrium analysis showed that the D’ and *r*^2^ values were 0.883 and 0.143, respectively.

In summary, the findings of our study provide further strong evidence that the *MTHFR* C677T and A1298C polymorphisms are usually in *trans* and occasionally in *cis* configurations. The frequencies of mutant genotype combinations were relatively higher in Chinese population than other populations, and showed geographical variations. These baseline data would be useful for future related studies and for developing health management programs.

## 1 Introduction

Methylenetetrahydrofolate reductase (MTHFR) is an enzyme that catalyzes the irreversible conversion of 5,10-methylenetetrahydrofolate to 5-methyltetrahydrofolate, the predominant circulatory form of folate in plasma and the methyl donor for the remethylation of homocysteine (Hcy) to methionine. The enzyme therefore resides at an important branch point directing the folate pool to Hcy remethylation and DNA methylation or to DNA and RNA biosynthesis.^[1]^ Genetic defects in *MTHFR* gene therefore have the potential to affect the disease risk both negatively and positively.^[1–3]^

Many polymorphisms in the *MTHFR* gene have been identified. Among them, C677T and A1298C are 2 common polymorphisms that have been confirmed to reduce the enzyme activity. The C677T polymorphism decreases the enzyme activity by 70% and 35% in homozygotes (TT) and heterozygotes (CT), respectively, and thus leads to increased plasma Hcy concentrations and abnormal DNA methylation status.^[4–6]^ The A1298C polymorphism can also reduce the enzyme activity but to a lesser extent than the C677T polymorphism.^[7]^ Neither the homozygous nor the heterozygous of the A1298C polymorphism is associated with elevated Hcy and/or lower folate concentrations. However, combined heterozygosity for the 2 polymorphisms, the 677CT/1298AC genotype, results in an even lower MTHFR activity than heterozygosity for either of the polymorphisms separately and results in elevated Hcy and decreased plasma folate levels as observed in homozygotes for the C677T polymorphism.^[8]^

During the past 2 decades, numerous epidemiological studies have investigated the relationships of the *MTHFR* C677T and A1298C polymorphisms with various diseases, including birth defects, cardiovascular diseases, pregnancy complications, lymphoblastic leukemia, rheumatoid arthritis, and breast cancer, although the results were still inconsistent.^[1,9–15]^ In addition, estimation of the genotype and haplotype distributions of the 2 polymorphisms in different populations has also been a focus of considerable interest from researchers worldwide. The 677T allele frequency is often reported to be high in Europeans and North Americans, low in East Asians and Africans, and also showing geographical gradients in some areas such as Europe, North America, and India.^[16–19]^ The 1298C allele was found with a high frequency in East Asia, followed by Europe, Africa, and North America.^[20]^ Our group previously recruited over 15,000 adults from 10 provinces to explore the geographical distribution of the *MTHFR* C677T and A1298C polymorphisms in China.^[21]^ We found that the prevalence of the 2 polymorphisms varied significantly among Han populations residing in different regions of China, and also showed apparent geographical gradients; the 677TT genotype frequencies steadily increased from southern to northern China, yet the 1298CC genotype frequencies showed a reverse geographical trend.^[21]^ The distribution of the least common mutant haplotype, 677T-1298C, also showed a large global variation: nil in Pakistan and Brazil, 2.6% in Mexico, 7.0% in Europe, and 0.5% in America.^[22]^

Prior studies have shown that there exists an interaction between the 2 polymorphisms in vivo, and that a combined effect of the 2 would result in clinical phenotypes and carry a selective disadvantage.^[8,23]^ Therefore, understanding the haplotype and combined genotype distribution of the 2 polymorphisms is important and valuable. Several case–control studies have reported the frequencies of the combined genotype and haplotype of the *MTHFR* C677T and A1298C polymorphisms in Chinese population,^[24–26]^ but most of these studies were limited by a focus on the prevalence at regional levels and/or small sample sizes. In this study, we estimated and reported the combined genotype and haplotype distributions of the *MTHFR* C677T and A1298C polymorphisms among 13,473 Chinese adult women from 9 provinces. These regions were distributed widely from south to north of China and have nearly half of Chinese population.

## 2 Methods

### 2.1 Study subjects

From October 2008 through February 2011, a total of 13,473 healthy unrelated women within the age group of 19 to 45 years (mean age, 27.7 ± 4.4 years) who came to local maternal and children's hospital for pre-pregnancy examination were enrolled in our study. These participants came from 9 provinces, which were located widely from southern to northern China. According to the divide represented by the Yangtze River, we further divided these individuals into 2 major groups: the northern and the southern. The northern group included participants from Shandong, Henan, and Shaanxi provinces; the southern group included those from Jiangsu, Hubei, Sichuan, Yunnan, Guangdong, and Hainan provinces. The study was conducted in accordance with the World Medical Association Declaration of Helsinki-Ethical Principles for Medical Research Involving Human Subjects, and all procedures were approved by the Ethics Committee of China Medical University (Shenyang, China; Identification code: CMU62073024; July 15, 2008). A written informed consent form was obtained from all participants before study entry. After obtaining due informed consent, buccal smears were collected, dried at room temperature for 1 hour, and then sent to the central laboratory in Shanghai.

### 2.2 Genotyping

Genomic DNA was extracted from buccal cells using the QIAamp DNA Mini Kit (Qiagen, Valencia, CA). We determined the *MTHFR* C677T and A1298C genotypes using the Taqman allelic discrimination assay on ABI 7900HT sequence detection system (Applied Biosystems, Foster City, CA) according to the manufacturer's instructions. All PCR reagents were purchased from ABI company. The detailed information on primers and probes was described in our previous paper.^[21]^ PCR amplification using about 5 ng/sample of genomic DNA was done in a thermal cycler (GeneAmp PCR Systeme 9700; Applied Biosystem, Foster City, CA). Cycling conditions were 95^o^C for 10 minutes, and 20 cycles of 92^o^C for 15 seconds and 60^o^C for 1 minute. Data analysis for allelic discrimination was performed using SDS software (Applied Biosystem, Foster City, CA).

### 2.3 Statistical analysis

Combined genotype frequencies were calculated by direct counting. Hardy–Weinberg equilibrium of the *MTHFR* polymorphisms in each population, and the difference in the prevalence of the combined genotype between the southern and northern groups were all examined by Chi-square test. The comparison of the combined genotype and haplotype frequencies among the 9 populations was examined using Kruskal–Wallis test. All the above analyses were performed using SAS version 9.2 (SAS Institute, Cary, NC). In addition, Haploview 4.1 software (Broad Institute, Cambridge, MA) was used to estimate haplotype frequencies and to perform linkage disequilibrium (LD) test. A 2-tailed *P* value < 0.05 was taken as statistically significant.

## 3 Results

### 3.1 Combined genotypes

The observed genotype frequencies of the *MTHFR* C677T and A1298C polymorphisms were all in accordance with Hardy–Weinberg equilibrium. Table 1 presents the frequencies of the combined *MTHFR* C677T and A1298C genotypes in the 9 Chinese Han populations. In the total sample, the 6 common combined genotypes were CT/AA (29.5%), TT/AA (21.9%), CC/AA (15.4%), CC/AC (14.9%), CT/AC (13.7%), and CC/CC (3.4%). In addition, we observed 51 (0.38%) individuals with the CT/CC genotype, 92 (0.7%) with the TT/AC genotype, and 17 (0.1%) with the TT/CC genotype. The distribution of the combined genotypes varied among the 9 populations (*P* < 0.001). For example, in Shandong province, the 3 most frequent combined genotypes in sequence were TT/AA (39.9%), CT/AA (28.6%), and CT/AC (15.7%); however, in Hainan province, they were CC/AC (26.6%), CC/AA (25.4%), and CT/AA (24.0%). Furthermore, we found that the prevalence of some combined genotypes showed apparent geographical gradients: the CC/AA, CC/AC, and CC/CC genotype frequencies steadily increased from northern to southern China, while the frequencies of the TT/AA genotype showed a reverse trend. After dividing the 9 populations into 2 groups, the northern group had significantly higher frequencies of the CT/AA (31.1% vs 27.8%), CT/AC (15.3% vs 12.1%), and TT/AA (44.0% vs 10.0%) genotypes, but lower frequencies of the CC/AA (8.7% vs 22.15%), CC/AC (8.2% vs 21.7%), and CC/CC (1.7% vs 5.2%) genotypes. The frequencies of the 3 rare genotypes (CT/CC, 0.4% vs 0.4%; TT/AC, 0.8% vs 0.6%; TT/CC, 0.1% vs 0.1%) were similar between the northern and southern groups.

**Table 1:**
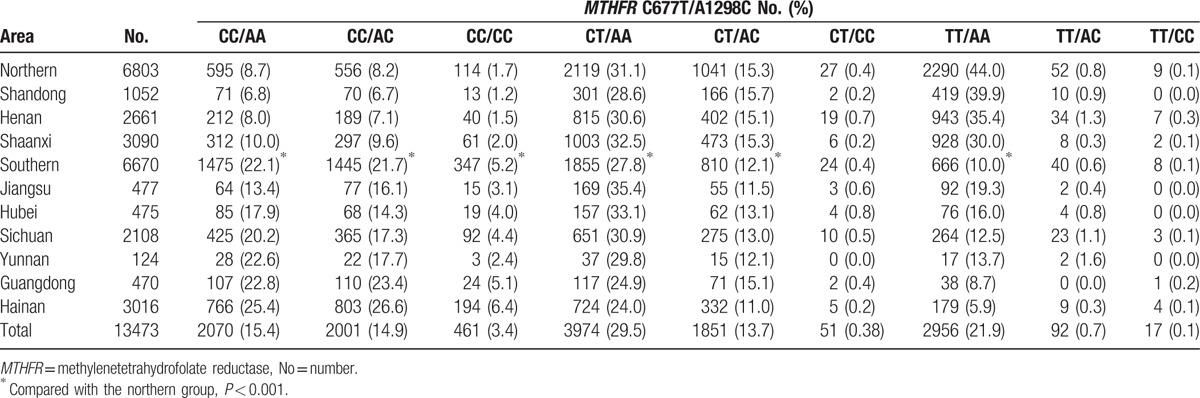
Frequency of combined methylenetetrahydrofolate reductase C677T and A1298C polymorphisms in populations from 9 provinces in China.

The allelic frequencies of the 2 polymorphisms also showed apparent geographical gradients. For example, the 677T allele frequency was lowest in Hainan province (24.0%, South), intermediate in Jiangsu province (43.5%, Central), and highest in Shandong province (63.1%, North). On the contrary, the 1298C allele frequency showed a decreasing trend from Hainan province (25.7%) to Shandong province (13.1%) (Table 2).

**Table 2:**
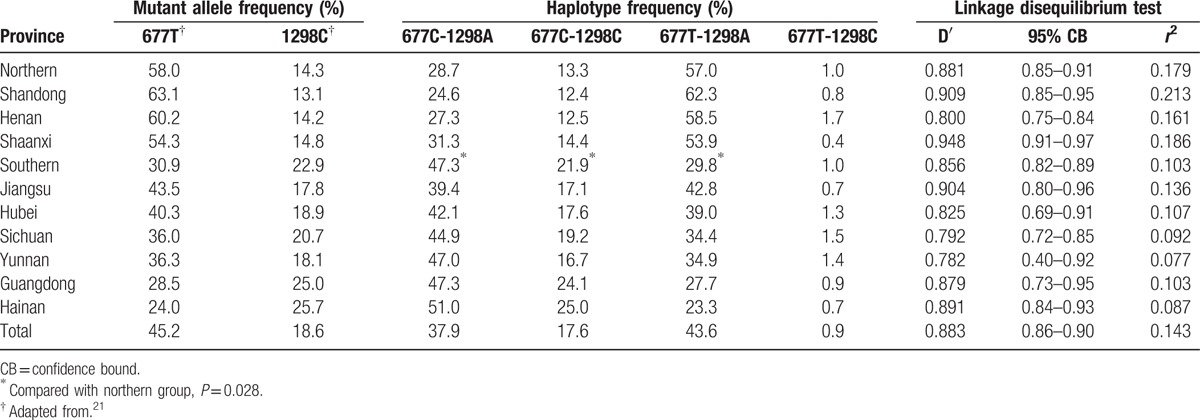
Estimated haplotype frequencies and results from linkage disequilibrium test of methylenetetrahydrofolate reductase C677T and A1298C polymorphisms in populations from 9 provinces in China.

### 3.2 Haplotypes

The LD analysis showed that the D’ and *r*^2^ values were 0.883 and 0.143, respectively, in the total sample, which indicates that the *MTHFR* C677T and A1298C polymorphisms were at high LD. Similar results were observed in the 9 populations with D’ values ranging from 0.782 to 0.909 and *r*^2^ values ranging from 0.077 to 0.213 (Table 2). We further performed haplotype analysis and found all the 4 haplotypes of the 2 polymorphisms. In the total sample, the most preponderant haplotype was 677T-1298A with a frequency of 43.6%, followed by 677C-1298A (37.9%), 677C-1298C (17.6%), and 677T-1298C (0.9%). The distribution of the haplotype frequencies also differed significantly among the 9 populations (*P* < 0.001). For example, Henan and Shaanxi provinces had the highest and lowest prevalences of the 677T-1298C haplotype (1.7% vs 0.4%), respectively. In addition, Shandong and Hainan provinces had the lowest and highest prevalences of the 677C-1298A (24.6% vs 51.0%) and the 677C-1298C haplotypes (12.4% vs 25.0%), and had the highest and lowest prevalences of the 677T-1298A haplotype (62.3% vs 23.3%), respectively. Geographically, the frequencies of the 677C-1298A and 677C-1298C haplotypes steadily increased from northern to southern China, whereas the frequencies of the 677T-1298A haplotype presented a reverse trend. The northern group had significantly higher frequency of the 677T-1298A haplotype (57.0% vs 29.8%), but lower frequencies of the 677C-1298A (28.7% vs 47.3%) and 677C-1298C (13.3% vs 21.9%) haplotypes. The frequencies of the 677T-1298C were similar between the 2 groups (1.0% vs 1.0%).

## 4 Discussion

In this study, we investigated the distribution of the combined genotypes and haplotypes of the *MTHFR* C677T and A1289C polymorphisms in a large sample of Chinese Han population. All 9 possible combined genotype combinations and 4 haplotypes were detected in the total sample. In addition, the genotypic and haplotypic frequencies varied among populations residing in different areas, and even showed apparent geographical gradients.

The common combined genotypes were CT/AA, TT/AA, CC/AA, CC/AC, CT/AC, and CC/CC, and the frequent haplotypes were 677T-1298A, 677C-1298A, and 677C-1298C, which were in agreement with the findings of other investigators.^[17,27,28]^ Among these common combined genotypes, the CT/AC genotype received the most attention because it was associated with lower enzyme activity and higher plasma Hcy concentrations than heterozygosity for either variant.^[7,8,29]^ The mean frequency of the CT/AC genotype in our study was 13.7%, which is similar to that of Mexicans, but lower than that of Turks, French, and Italians, and higher than that of Africans.^[30–33]^ The CT/CC, TT/AC, and TT/CC genotypes were often reported to be rare combinations. Initially, several studies did not find any individual with the CT/CC, TT/AC, or TT/CC genotypes, thus concluding that the 2 mutant alleles were always in *trans* configuration.^[8,34,35]^ However, subsequent studies casted doubt on these speculations. Weisberg et al^[7]^ detected 1 child with spina bifida who carried TT/AC genotype; a meta-analysis of 22 studies with 12,647 subjects found 31 individuals (0.25%) with the CT/CC genotype, 58 (0.46%) with the TT/AC genotype, 4 four (0.03%) with the TT/CC genotype^[28]^; the most recent study conducted in India observed that the frequency of TT/AC genotype plus CT/CC genotype was 0.94%, and the 677T-1298C haplotype frequencies ranged from 1.2% to 3.6% in different population groups.^[17]^ In this study, we observed that 51 individuals were the CT/CC genotype, 92 were the TT/AC genotype, 17 were TT/CC genotype, and that the frequency of 677T-1298C haplotype was 0.9%. These findings indicate that although the *MTHFR* C677T and A1298C polymorphisms are usually in *trans*, they are occasionally in *cis* configurations, and indicate that the 2 polymorphisms are at incomplete LD, which is further substanticated by a formal LD test in our study.

One explanation for the absence or low prevalence of the CT/CC, TT/AC, and TT/CC genotypes in our and previous studies is the physical distance that separates the *MTHFR* C677T and A1298C polymorphisms on the chromosome is short (2.1 kb), which may reduce the probability of a recombinant event.^[35,36]^ Another possible explanation, as theorized by van der Put et al,^[8]^ is that the occurrence of the 3 genotype combinations, which allows 3 or more mutant alleles to be existed in the genome of one individual, could result in a selection disadvantage because of the expression of severe phenotypes. Despite the fact that the participants carrying the CT/CC, TT/AC, and TT/CC genotype combinations in our and some previous studies are healthy persons without any severe disease,^[17,28]^ we can still not deny the theory proposed by van der Put et al^[8]^ because the development of 1 disease is influenced by environmental and genetic factors as well as their interactions. Moreover, it has been suggested that B vitamins (especially folate) intake can neutralize the effect of the *MTHFR* mutant alleles.^[11]^ Thus, presumably the survival of the individuals carrying the 3 rare genotype combinations may have benefited from sufficient B vitamins fortification. As the CT/CC, TT/AC, and TT/CC genotype combinations is rare, little information is available on their relationships with Hcy and other clinical disorders. However, we should keep in mind that approximately 1.2% (160/13473) of the total population in our study carried the CT/CC or TT/AC or TT/CC genotype. Although the prevalence is relatively low, it is estimated that there will be a substantial number of people who carry these potentially deleterious genotype combinations due to an immense population base in China. Further well-designed studies therefore are still needed to fully explore their effects on clinical conditions. From a perspective of public health, genetic testing and prevention strategies (such as B vitamins fortification) based on genetic information for specific populations are needed in order to reduce the risk of diseases closely related to the mutant genotype combinations (e.g., birth defects).

Another important finding of the present study is that the common combined genotype and haplotype frequencies showed geographical gradients. For example, the frequencies of the CC/AA, CC/AC, and CC/CC genotypes steadily increased from northern to southern China, but the TT/AA frequencies showed a reverse trend. Correspondingly, the frequencies of 677C-1298A, 677C-1298C increased from northern to southern China, while the 677T-1298A haplotype frequencies increased in roughly northern direction. The geographical distribution of the *MTHFR* polymorphisms, especially the C677T polymorphism, has been investigated in many populations worldwide. Consistent with our findings, a south to north cline of increase in the 677T allele frequency was reported among Indians and Pakistanis.^[17,37]^ However, a reverse trend was observed in European and North America populations.^[18,19]^ The reasons for these geographical gradients remain unclear because many factors, including migratory histories, nutrients intake (especially folic acid, vitamin B_2_, and vitamin B_12_), disease, and environmental exposures (such as ultroviolet radiation), may play a role in the propagation of the 2 alleles.^[21,38]^

Numerous studies have explored the independent association of the *MTHFR* C677T or A1298C polymorphism with various clinical conditions.^[1,9–15]^ The results showed that the 2 polymorphisms can affect diseases risks both positively (such as neural tube defects, coronary heart disease, hypertension, and several cancers)^[1,9,10,15]^ and negatively (such as colorectal, colon, and prostate cancers).^[3,39]^ The relationship between the geographical variations of the C677T polymorphism and the prevalence of related diseases is complicated in China. For example, the prevalence rates of neural tube defects and hypertension increased from south to north region,^[40,41]^ a trend that follows the 677T allele frequencies, and also the TT/AA genotype and 677T-1298A haplotype frequencies. However, there are some exceptions. In the southern China, the prevalence rates of Alzheimer disease and nasopharyngeal cancer were high,^[42,43]^ but the 677T allele was infrequent. With respect to the A1298C polymorphism, although it was not as extensively studied as the C677T polymorphism, epidemiological studies have linked the 1298AA genotype to an increased risk of several diseases such as lymphoblastic leukemia and rheumatoid arthritis.^[12–14]^ The findings remind us that not only the C677T polymorphism but also the A1298C polymorphism should warrant more attention, especially considering that in our present study, the combined TT/AA is the most represented in northern China, while CT/AA is most represented in the southern China. Also, this may partially explain the inconsistencies between geographical distribution of the C677T polymorphism and prevalence of related diseases mentioned above, because the development of the 677T allele related diseases could also be affected by environmental factors and other polymorphisms, especially the A1298C polymorphism. Nevertheless, our data on the geographical distribution of the combined genotype and haplotype may be helpful for researchers seeking to investigate such relationships.

In interpreting the findings of this study, some limitations and strengths should be considered. One limitation of our study was that all study subjects are childbearing age women, which limits the generalizability to men and other age groups. However, no significant gender difference in distribution of the 2 polymorphisms was observed in our and other published studies.^[21]^ Another limitation was that the sample sizes from some provinces were small and many regions of China were not covered, which could have compromised our estimates. A major strength of this study was that our estimates, including D’ and *r*^2^ statistics, combined genotype and haplotype frequencies, were based on a substantial number of Chinese population, which could be used as authoritative reference data.

In conclusion, this study detected relatively higher frequencies of mutant genotype combinations in Chinese population, demonstrated significant geographical variations in the prevalence of combined genotypes and haplotypes of the *MTHFR* C677T and A198C polymorphisms, and reconfirmed that the 2 polymorphisms are usually in *trans* and occasionally in *cis* configurations. Because the 2 polymorphisms and their combinations are closely related to many disorders, including birth defects, vascular and neurodegenerative diseases, cancers, and pregnancy complications, our study provided important baseline data for future genetic association studies, and would be useful for government and health experts to develop regional health management programs.

## Acknowledgment

We gratefully acknowledge the assistance and cooperation of the faculty and staff of the local maternal and children's hospital and thank all of the participants in our study.

## References

[1] Ueland PM Rozen R MTHFR Polymorphisms and Disease. 1st ed. Landes Bioscience: Texas; 2005.

[2] Zhu XL Liu ZZ Yan SX Association between the *MTHFR* A1298C polymorphism and risk of cancer: evidence from 265 case-control studies. Mol Genet Genomics 2016;291:51–63. 26156333 10.1007/s00438-015-1082-y

[3] Xie SZ Liu ZZ Yu JH Association between the *MTHFR* C677T polymorphism and risk of cancer: evidence from 446 case-control studies. Tumour Biol 2015;36:8953–72. 26081619 10.1007/s13277-015-3648-z

[4] Frosst P Blom HJ Milos R A candidate genetic risk factor for vascular disease: a common mutation in methylenetetrahydrofolate reductase. Nat Genet 1995;10:111–3. 7647779 10.1038/ng0595-111

[5] Jacques PF Bostom AG Williams RR Relation between folate status, a common mutation in methylenetetrahydrofolate reductase, and plasma homocysteine concentrations. Circulation 1996;93:7–9. 8616944 10.1161/01.cir.93.1.7

[6] Weiner AS Boyarskikh UA Voronina EN Methylenetetrahydrofolate reductase C677T and methionine synthase A2756G polymorphisms influence on leukocyte genomic DNA methylation level. Gene 2014;533:168–72. 24103477 10.1016/j.gene.2013.09.098

[7] Weisberg I Tran P Christensen B A second genetic polymorphism in methylenetetrahydrofolate reductase (*MTHFR*) associated with decreased enzyme activity. Mol Genet Metab 1998;64:169–72. 9719624 10.1006/mgme.1998.2714

[8] van der Put NM Gabreels F Stevens EM A second common mutation in the methylenetetrahydrofolate reductase gene: an additional risk factor for neural-tube defects? Am J Hum Genet 1998;62:1044–51. 9545395 10.1086/301825 PMC1377082

[9] Liew SC Gupta ED Methylenetetrahydrofolate reductase (*MTHFR*) C677T polymorphism: epidemiology, metabolism and the associated diseases. Eur J Med Genet 2015;58:1–0. 25449138 10.1016/j.ejmg.2014.10.004

[10] Yang B Fan S Zhi X Associations of *MTHFR* gene polymorphisms with hypertension and hypertension in pregnancy: a meta-analysis from 114 studies with 15411 cases and 21970 controls. PLoS One 2014;9:e87497. 24505291 10.1371/journal.pone.0087497 PMC3914818

[11] Moll S Varga EA Homocysteine and *MTHFR* mutations. Circulation 2015;132:e6–9. 26149435 10.1161/CIRCULATIONAHA.114.013311

[12] Mosaad YM Abousamra NK Elashery R Methylenetetrahydrofolate reductase C677T and A1298C polymorphism and susceptibility to acute lymphoblastic leukemia in a cohort of Egyptian children. Leuk Lymphoma 2015;56:2699–705. 25629981 10.3109/10428194.2015.1004170

[13] Bahari G Hashemi M Naderi M Association between methylenetetrahydrofolate reductase (MTHFR) gene polymorphisms and susceptibility to childhood acute lymphoblastic leukemia in an Iranian population. Int J Hematol Oncol Stem Cell Res 2016;10:130–7. 27489588 PMC4969557

[14] Cen H Huang H Zhang LN Associations of methylenetetrahydrofolate reductase (MTHFR) C677T and A1298C polymorphisms with genetic susceptibility to rheumatoid arthritis: a meta-analysis. Clin Rheumatol 2016;[Epub ahead of print]. DOI: 10.1007/s10067-016-3348-0. 10.1007/s10067-016-3348-0 27423206

[15] Zhong S Chen Z Yu X A meta-analysis of genotypes and haplotypes of methylenetetrahydrofolate reductase gene polymorphisms in breast cancer. Mol Biol Rep 2014;41:5775–85. 24973876 10.1007/s11033-014-3450-9

[16] Kidd K ALFRED: the ALlele FREquency Databese. Available at: http://alfred.med.yale.edu/alfred/SiteTable1A_working.asp?siteuid=SI001032G Accessed April 26, 2015.

[17] Saraswathy KN Asghar M Samtani R Spectrum of *MTHFR* gene SNPs C677T and A1298C: a study among 23 population groups of India. Mol Biol Rep 2012;39:5025–31. 22147263 10.1007/s11033-011-1299-8

[18] Pepe G Camacho VO Giusti B Heterogeneity in world distribution of the thermolabile C677T mutation in 5,10-methylenetetrahydrofolate reductase. Am J Hum Genet 1998;63:917–20. 9718345 10.1086/302015 PMC1377403

[19] Wilcken B Bamforth F Li Z Geographical and ethnic variation of the 677C>T allele of 5,10 methylenetetrahydrofolate reductase (*MTHFR*): findings from over 7000 newborns from 16 areas worldwide. J Med Genet 2003;40:619–25. 12920077 10.1136/jmg.40.8.619 PMC1735571

[20] Kidd K ALFRED: the ALlele FREquency Databese. Available at: http://alfred.med.yale.edu/alfred/SiteTable1A_working.asp?siteuid=SI003687Y Accessed April 26, 2015.

[21] Yang B Liu Y Li Y Geographical distribution of *MTHFR* C677T, A1298C and *MTRR* A66G gene polymorphisms in China: findings from 15357 adults of Han nationality. PLoS One 2013;8:e57917. 23472119 10.1371/journal.pone.0057917 PMC3589470

[22] Shi M Caprau D Romitti P Genotype frequencies and linkage disequilibrium in the CEPH human diversity panel for variants in folate pathway genes *MTHFR*, *MTHFD*, *MTRR*, *RFC1*, and *GCP2*. Birth Defects Res A Clin Mol Teratol 2003;67:545–9. 14632302 10.1002/bdra.10076

[23] Isotalo PA Wells GA Donnelly JG Neonatal and fetal methylenetetrahydrofolate reductase genetic polymorphisms: an examination of C677T and A1298C mutations. Am J Hum Genet 2000;67:986–90. 10958762 10.1086/303082 PMC1287901

[24] Qu HH Cui LH Wang K The methylenetetrahydrofolate reductase C677T polymorphism influences risk of esophageal cancer in Chinese. Asian Pacific J Cancer Prev 2013;14:3163–618. 10.7314/apjcp.2013.14.5.3163 23803097

[25] Lin J Zeng RM Li RN Aberrant DNA methylation of the P16, MGMT, and hMLH1 genes in combination with the methylenetetrahydrofolate reductase C677T genetic polymorphism and folate intake in gastric cancer. Genet Mol Res 2014;13:2060–8. 24737431 10.4238/2014.March.24.10

[26] Lv QQ Lu J Sun H Association of methylenetetrahydrofolate reductase (MTHFR) gene polymorphism with ischemic stroke in the Eastern Chinese Han population. Genet Mol Res 2015;14:4161–8. 25966188 10.4238/2015.April.27.31

[27] Ulvik A Ueland PM Fredriksen A Functional inference of the methylenetetrahydrofolate reductase 677C > T and 1298A > C polymorphisms from a large-scale epidemiological study. Hum Genet 2007;121:57–64. 17115185 10.1007/s00439-006-0290-2

[28] Ogino S Wilson RB Genotype and haplotype distributions of *MTHFR* 677C>T and 1298A>C single nucleotide polymorphisms: a meta-analysis. J Hum Genet 2003;48:1–7. 12560871 10.1007/s100380300000

[29] Dekou V Whincup P Papacosta O The effect of the C677T and A1298C polymorphisms in the methylenetetrahydrofolate reductase gene on homocysteine levels in elderly men and women from the British regional heart study. Atherosclerosis 2001;154:659–66. 11257267 10.1016/s0021-9150(00)00522-0

[30] Sazci A Ergul E Kaya G Genotype and allele frequencies of the polymorphic methylenetetrahydrofolate reductase gene in Turkey. Cell Biochem Funct 2005;23:51–4. 15386535 10.1002/cbf.1132

[31] Zappacosta B Romano L Persichilli S Genotype prevalence and allele frequencies of 5,10-methylenetetrahydrofolate reductase (*MTHFR*) C677T and A1298C polymorphisms in Italian newborns. Labmedicine 2009;12:732–6.

[32] Esfahani ST Cogger EA Caudill MA Heterogeneity in the prevalence of methylenetetrahydrofolate reductase gene polymorphisms in women of different ethnic groups. J Am Diet Assoc 2003;103:200–7. 12589326 10.1053/jada.2003.50030

[33] Falchi A Giovannoni L Piras IS Prevalence of genetic risk factors for coronary artery disease in Corsica Island (France). Exp Mol Pathol 2005;79:210–3. 16248996 10.1016/j.yexmp.2005.09.005

[34] Rady PL Tyring SK Hudnall SD Methylenetetrahydrofolate reductase (*MTHFR*): the incidence of mutations C677T and A1298C in the Ashkenazi Jewish population. Am J Med Genet 1999;86:380–4. 10494095 10.1002/(sici)1096-8628(19991008)86:4<380::aid-ajmg13>3.0.co;2-9

[35] Stegmann K Ziegler A Ngo ET Linkage disequilibrium of *MTHFR* genotypes 677C/T-1298A/C in the German population and association studies in probands with neural tube defects (NTD). Am J Med Genet 1999;87:23–9. 10528242

[36] Hanson NQ Aras O Yang F C677T and A1298C polymorphisms of the methylenetetrahydrofolate reductase gene: incidence and effect of combined genotypes on plasma fasting and post-methionine load homocysteine in vascular disease. Clin Chem 2001;47:661–6. 11274015

[37] Mansoor A Mazhar K Ali L Prevalence of the C677T single-nucleotide polymorphism in the methylenetetrahydrofolate reductase gene among Pakistani ethnic groups. Genet Test Mol Biomarkers 2009;13:521–6. 19594369 10.1089/gtmb.2009.0012

[38] Wang YF Pei LJ Wang JF Is the prevalence of *MTHFR* C677T polymorphism associated with ultraviolet radiation in Eurasia? J Hum Genet 2012;57:780–6. 22992775 10.1038/jhg.2012.113

[39] Safarinejad MR Shafiei N Safarinejad S Relationship between three polymorphisms of methylenetetrahydrofolate reductase (*MTHFR* C677T, A1298C, and G1793A) gene and risk of prostate cancer: a case-control study. Prostate 2010;70:1645–57. 20564317 10.1002/pros.21200

[40] Li Z Ren A Zhang L Extremely high prevalence of neural tube defects in a 4-country area in Shanxi Province, China. Birth Defects Res A Clin Mol Teratol 2006;76:237–40. 16575897 10.1002/bdra.20248

[41] Zhao L Stamler L Yan LL Blood pressure differences between northern and southern Chinese: role of dietary factors: the International Study on Macronutrients and Blood Pressure. Hypertension 2004;43:1332–7. 15117915 10.1161/01.HYP.0000128243.06502.bc PMC6688605

[42] Dong MJ Peng B Lin XT The prevalence of dementia in the People's Republic of China: a systematic analysis of 1980–2004 studies. Age Ageing 2007;36:619–24. 17965036 10.1093/ageing/afm128

[43] Yu MC Yuan JM Epidemiology of nasopharyngeal carcinoma. Semin Cancer Biol 2002;12:421–9. 12450728 10.1016/s1044579x02000858

